# Editorial: Infection, inflammation, and neurodegeneration: A critical path to Alzheimer's disease, Volume II

**DOI:** 10.3389/fnagi.2022.1044047

**Published:** 2022-10-06

**Authors:** Roberta Mancuso, Simone Agostini, Denah M. Appelt, Brian J. Balin

**Affiliations:** ^1^IRCCS Fondazione Don Carlo Gnocchi ONLUS, Milan, Italy; ^2^Philadelphia College of Osteopathic Medicine, Philadelphia, PA, United States

**Keywords:** infection, inflammation, neurodegeneration, Alzheimer's disease, aging

Alzheimer's disease (AD) is the most prevalent cause of dementia, that represents one of the major health challenges for humanity, with significant social and economic implications. This complex and progressive neurodegenerative disorder is characterized by specific molecular hallmarks, as amyloid beta deposition and neurofibrillary tau tangles. The etiology of this disease is far from being fully understood, and the possibility of an infectious cause has long been debated.

Even Alois Alzheimer, himself, over 100 years ago studied *Treponema pallidum*, the causative agent of syphilis, a spirochete later associated with dementia (Noguchi and Moore, 1913). Oskar Fischer, a contemporary of Alzheimer and also a psychiatrist and neuropathologist, also suggested that microorganisms could form plaques in brain tissues (Fischer, 1907). Sporadic late-onset AD, accounting for ~95–98% of all cases of AD is thought to arise due to a multi-factorial interplay between genetic and environmental factors. Linkage has been identified to risk factors including ApoEε4 expression, chronic neuroinflammation, autoimmune mechanisms, oxidative and mitochondrial damage, cardiovascular factors, diabetes with insulin resistance, trauma to the blood brain barrier and selectively vulnerable brain insult. Thus, infection actually may be the overarching “unifying hypothesis” for sporadic late-onset AD, rather than other more mainstream hypotheses.

This Research Topic represents the efforts of the scientific community to better understand causative factors for sporadic late-onset AD, and lends itself to consider multiple elements and mechanisms participating in the pathogenesis of AD.

Among many factors that may drive the accumulation of amyloid and tau in AD, infectious triggers are some of the most significant and logical choices. In particular, the organisms likely to be involved in AD are those that can evade host immune defenses, gain entry to specific selectively vulnerable regions of the brain, and establish chronic/persistent and/or latent infection. Early studies of infection did not find in AD brains the direct evidence of viral nucleic acid sequences and antigens of cytomegalovirus, measles virus, poliovirus, adenoviruses, hepatitis B virus, and the influenza A and B viruses (Pogo et al., 1987), although other authors have shown the presence of HSV-1 (Ball, 1982; Itzhaki et al., 1997), *Borrelia burgdorferi* (Miklossy, 1993), and *Chlamydia pneumoniae* (Balin et al., 1998; Gerard et al., 2006) in AD brains. Besides that, over the years the hypothesis on the possible role of HSV-1 in AD has been supported by several *in vitro* studies, suggesting that HSV-1 infection and its multiple reactivation can trigger oxidative damage and progressive accumulation of neurotoxic products (Amyloid-beta and hyperphosphorylated Tau aggregates), leading to neurodegeneration and cognitive decline (see: Marcocci et al., 2020). Moreover, host immunity and its decline with aging can have an effect on the control of HSV-1 viral reactivation from latency. Epidemiological studies of the IgG avidity index as an indicator of HSV-1 reactivation and the relationships between HSV-1-specific humoral immunity and cortical damage measured by MRI, even in relation with the host genetic pattern (see: Mancuso et al., 2019), allow one to hypothesize the possible protective role of antiviral IgG, although it remains to be confirmed by others.

Moreover, in the last twenty years, other different types of infections have been associated with dementing illnesses, including infection with *Treponema pallidum* (Miklossi, 2011), as well as *Cryptococcus neoformans* (Hoffmann et al., 2009), measles virus (Frings et al., 2002), and HIV (Zhou et al., 2010; Canet et al., 2018). More recently, several studies have correlated SARs-CoV2 virus in furthering the pathogenesis of AD (Fu et al., 2022). Intriguingly, the involvement of the microbiota in AD pathophysiology has also been gaining attention, as it may be a key factor influencing brain function; intestinal microbiota seems to interfere with Abeta assembly in brain (Wang et al., 2015), but even oral microbiota can play a role in onset and progression of AD (Sureda et al., 2020).

Local periodontal inflammation due to infection with *Helicobacter pylori* (Beydoun et al., 2021), the agent of gastric ulcers, or with *Porphyromonas gingivalis* (Elwishahy et al., 2021), in fact, can stimulate brain tissue inflammation (Dominy et al., 2019). Moreover, Wu et al. provided evidence *in vitro* that *Fusobacterium nucleatum* can increased TNF-α and IL-1β expression in microglial cells, and also *in vivo* it can increase cognitive impairment, beta-amyloid accumulation and Tau protein phosphorylation in the cerebrum of an AD animal model.

The impact of systemic acute infections on neurodegeneration has been previously evaluated in animal models of chronic neurodegeneration (Cunningham et al., 2009), but only limited data exist on humans. The study of Silva et al. analyzed the neuroinflammatory response triggered by acute systemic inflammation in older subjects with dementia compared to cognitively healthy controls. Using PET imaging, the authors showed that the level of neuroinflammation due to acute systemic infection was reduced in subjects with dementia and/or delirium compared to cognitively healthy participants. Future investigations are warranted to confirm these observations, in order to clarify if “dementia” with changes in brain has a distinct neuroinflammatory profile possibly owing to immunosuppression within the brain in response to acute systemic infection.

Lei et al. conducted a systematic review and meta-analysis to determine the relationship between sepsis survivals and risk of dementia in elderly individuals. They determined that there was an increased risk of all-cause dementia in sepsis survival subjects suggesting the importance of an appropriate management and prevention scheme to preserve cognitive function.

Finally, Nelson explored the hypothesis that infections can contribute to AD through the generation of peripheral amyloids and/or neurovascular dysfunction. After a careful analysis of available data supporting the neurovascular dysfunction and blood-brain barrier permeability in AD, the author proposes in this interesting review a new “peripheral amyloid hypothesis” as a possible actor leading to cognitive impairment.

In summary, we are very grateful to the researchers that contributed with an article as well the reviewers who have contributed with their reviews to the quality of this Research Topic. Overall, this Research Topic and the entire contribution of literature to this hypothesis enables us to consider how infections, and consequently the host immune response, may play a significant role in the etiology of AD. We contend that consideration of infection and subsequent inflammation in the pathogenesis of AD may provide new avenues for intervention to both delay progression and, ultimately, prevent disease.

## Author contributions

All authors listed have made a substantial, direct and intellectual contribution to the work, and approved it for publication.

## Conflict of interest

The authors declare that the research was conducted in the absence of any commercial or financial relationships that could be construed as a potential conflict of interest.

## Publisher's note

All claims expressed in this article are solely those of the authors and do not necessarily represent those of their affiliated organizations, or those of the publisher, the editors and the reviewers. Any product that may be evaluated in this article, or claim that may be made by its manufacturer, is not guaranteed or endorsed by the publisher.

## References

[B1] Balin B. J. Gerard H. C. Arking E. J. Appelt D. M. Branigan P. J. Abrams J. T.. (1998). Identification and localization of *Chlamydia pneumoniae* in the Alzheimer's brain. Med. Microbiol. Immunol. 187, 23–42. 10.1007/s004300050071 9749980

[B2] Ball M. J. (1982). Limbic predilection in Alzheimer dementia: is reactivated herpesvirus involved? Can. J. Neurol. Sci. 9, 303–306. 10.1017/S0317167100044115 7116237

[B3] Beydoun M. A. Beydoun H. A. Weiss J. Hossain S. El-Hajj Z. W. Zonderman A. B. (2021). *Helicobacter pylori*, periodontal pathogens, and their interactive association with incident all-cause and Alzheimer's disease dementia in a large national survey. Mol. Psychiatry. 26, 6038–6053. 10.1038/s41380-020-0736-2 32366948

[B4] Canet G. Dias C. Gabelle A. Simonin Y. Gosselet F. Marchi N.. (2018). HIV neuroinfection and Alzheimer's disease: similarities and potential links? Front. Cell. Neurosci. 12, 307. 10.3389/fncel.2018.00307 30254568 PMC6141679

[B5] Cunningham C. Campion S. Lunnon K. Murray C. L. Woods J. F. Deacon R. M.. (2009). Systemic inflammation induces acute behavioral and cognitive changes and accelerates neurodegenerative disease. Biol. Psychiatry. 65, 304–312. 10.1016/j.biopsych.2008.07.024 18801476 PMC2633437

[B6] Dominy S. S. Lynch C. Ermini F. Benedyk M. Marczyk A. Konradi A.. (2019). Porphyromonas gingivalis in Alzheimer's disease brains: evidence for disease causation and treatment with small-molecule inhibitors. Sci. Adv. 5:eaau3333 10.1126/sciadv.aau3333 30746447 PMC6357742

[B7] Elwishahy A. Antia K. Bhusari S. Ilechukwu N. C. Horstick O. Winkler V. (2021). Porphyromonas gingivalis as a risk factor to alzheimer's disease: a systematic review. J. Alzheimers. Dis. Rep. 5, 721–732. 10.3233/ADR-200237 34755046 PMC8543378

[B8] Fischer O. (1907). Miliare Nekrosen mit drusigen Wucherungen der Neurofibrillen, eine regelmässige Veränderung der Hirnrinde bei seniler Demenz. Monatschr. F. Psychiat. Neurol. 22, 361–372. 10.1159/000211873

[B9] Frings M. Blaeser I. Kastrup O. (2002). Adult-onset subacute sclerosing panencephalitis presenting as a degenerative dementia syndrome. J. Neurol. 249, 942–943. 10.1007/s00415-002-0720-6 12212560

[B10] Fu Y. W. Xu H. S. Liu S. Y. (2022). COVID-19 and neurodegenerative diseases. Eur. Rev. Med. Pharmacol. Sci. 26, 4535–4544. 10.26355/eurrev_202206_29093 35776055

[B11] Gerard H. C. Dreses-Werringloer U. Wildt K. S. Deka S. Oszust C. Balin B. J.. (2006). Chlamydophila (Chlamydia) pneumoniae in the Alzheimer's brain. FEMS Immunol. Med. Microbiol. 48, 355–366. 10.1111/j.1574-695X.2006.00154.x 17052268

[B12] Hoffmann M. Muniz J. Carroll E. De Villasante J. (2009). Cryptococcal meningitis misdiagnosed as Alzheimer's disease: complete neurological and cognitive recovery with treatment. J. Alzheimers. Dis. 16, 517–520. 10.3233/JAD-2009-0985 19276545

[B13] Itzhaki R. F. Shang D. Wilcock G. K. Faragher B. Jamieson G. A. (1997). Herpes simplex virus type 1 in brain and risk of Alzheimer's disease. Lancet. 349, 241–244. 10.1016/S0140-6736(96)10149-5 9014911

[B14] Mancuso R. Sicurella M. Agostini S. Marconi P. (2019). Herpes simplex virus type 1 and Alzheimer's disease: link and potential impact on treatment. Expert Rev. Anti. Infect. Ther. 17, 715–731. 10.1080/14787210.2019.1656064 31414935

[B15] Marcocci M. E. Napoletani G. Protto V. Kolesova O. Piacentini R. Li Puma D. D.. (2020). Herpes simplex virus-1 in the brain: the dark side of a sneaky infection. Trends Microbiol. 28, 808–820. 10.1016/j.tim.2020.03.003 32386801

[B16] Miklossi J. (2011). Alzheimer's disease – a neurospirochetosis. Analysis of the evidence following Koch's and Hill's criteria. J. Neuroinflammation. 8, 90. 10.1186/1742-2094-8-90 21816039 PMC3171359

[B17] Miklossy J. (1993). Alzheimer's disease–a spirochetosis? Neuroreport. 4, 841–848. 10.1097/00001756-199307000-00002 8369471

[B18] Noguchi H. Moore J. W. (1913). A demonstration of treponema pallidum in the brain in cases of general paralysis. J. Exp. Med. 17, 232–238. 10.1084/jem.17.2.232 19867640 PMC2125022

[B19] Pogo B. G. Casals J. Elizan T. S. (1987). A study of viral genomes and antigens in brains of patients with Alzheimer's disease. Brain. 110, 907–915. 10.1093/brain/110.4.907 2443213

[B20] Sureda A. Daglia M. Argüelles Castilla S. Sanadgol N. Fazel Nabavi S. Khan H.. (2020). Oral microbiota and Alzheimer's disease: do all roads lead to Rome? Pharmacol. Res. 151, 104582. 10.1016/j.phrs.2019.104582 31794871

[B21] Wang D. Ho L. Faith J. Ono K. Janle E. M. Lachcik P. J.. (2015). Role of intestinal microbiota in the generation of polyphenol-derived phenolic acid mediated attenuation of Alzheimer's disease β-amyloid oligomerization. Mo.l Nutr. Food. Res. 59, 1025–1040. 10.1002/mnfr.201400544 25689033 PMC4498582

[B22] Zhou L. Diefenbach E. Crossett B. Tran S. L. Ng T. Rizos H.. (2010). First evidence of overlaps between HIV-Associated Dementia (HAD) and non-viral neurodegenerative diseases: proteomic analysis of the frontal cortex from HIV+ patients with and without dementia. Mol. Neurodegen. 5, 27. 10.1186/1750-1326-5-27 20573273 PMC2904315

